# Non-targeted metabolomics reveals tissue-specific metabolic profiling and antioxidant - related markers in *Mosla chinensis* Maxim. cv. Jiangxiangru

**DOI:** 10.3389/fpls.2026.1740266

**Published:** 2026-02-18

**Authors:** Yuling Wang, Jiale Zhang, Mengxing Wang, Jianfeng Cheng, Wuping Yan

**Affiliations:** 1 School of Agricultural Sciences, Jiangxi Agricultural University, Nanchang, China; 2 Jiangxi Province Key Laboratory of Vegetable Cultivation and Utilization, Jiangxi Agricultural University, Nanchang, China

**Keywords:** antioxidant, flavonoid, *Mosla chinensis* Maxim. cv. Jiangxiangru (McJXR), phenol, tissue

## Abstract

*Mosla chinensis* Maxim. cv. Jiangxiangru (McJXR) is an aromatic medicinal plant, but its tissue-specific metabolites and antioxidant capacity lack systematic characterization. This study utilized non-targeted metabolomics to comprehensively characterize the metabolic profiles of the stem, leaf, and flower tissues of McJXR, and to assess their *in vitro* antioxidant activities. A total of 87 compounds were identified by GC–MS, among which 12, 6, and 8 were uniquely present in the flower, stem, and leaf, respectively. LC–MS/MS detected 21,636 and 21,880 ion features in positive and negative ion modes, respectively, revealing a distinct tissue-specific distribution of metabolites, with 176, 169 and 154 metabolites identified in the flower, stem and leaf, respectively. The OPLS-DA model (positive ion mode: R²X = 0.684, Q² = 0.962) further confirmed the reliability of metabolic profile differences across tissues. The differential metabolites (DMs) were primarily enriched in the ABC transporter pathway and the biosynthesis pathways of plant secondary metabolites. Antioxidant activity followed the order: flower > leaf > stem. Total flavonoid content (TFC, reaching up to 10.82 mg RE/g DW in the flower) and total phenolic content (TPC, peaking at 99.25 mg GAE/g DW in the leaf) exhibited significant positive correlations with antioxidant capacity (r > 0.5, p < 0.05), with TFC showing a stronger correlation (r = 0.9443 with the ABTS assay, p < 0.01). Mantel test results identified octopamine, 4-aminobutyric acid, and xylitol as potential antioxidant-related metabolites. This study elucidated the tissue-specific distribution patterns of McJXR metabolites and the biochemical basis of their antioxidant activity, providing a scientific foundation for the rational selection of medicinal parts and the development of functional constituents.

## 1 Introduction

Traditional Chinese medicine and natural products represent not only vital sources for modern drug discovery and development, but also key strategic resources for China in the areas of public health security, economic growth, and sustainable utilization of ecological assets (Ma et al., 2022). These resources play an indispensable role in the innovation and advancement of novel therapeutics. Numerous bioactive compounds derived from medicinal plants have been successfully developed into first-line clinical drugs. For example, artemisinin, a sesquiterpene lactone isolated from *Artemisia Annua*, is globally recognized and widely applied as a core antimalarial agent (Meng et al., 2021); tanshinones, the principal lipophilic bioactive constituents of *Salvia miltiorrhiza*, exhibit a range of pharmacological activities, including enhancement of coronary circulation, inhibition of platelet aggregation, anti-inflammatory, antioxidant, and anticancer effects (Song et al., 2022); taxol, a rare diterpenoid compound extracted from *Taxus* species, is extensively used in the clinical management of various malignancies, such as breast and ovarian cancers (Li et al., 2019); morphine, an isoquinoline alkaloid derived from *Papaver Somniferum*, continues to serve as one of the most potent analgesic and anesthetic agents in contemporary medical practice (Guo et al., 2018); patchoulol, a tricyclic sesquiterpene alcohol derived from *Pogostemon cablin*, exhibits notable pharmacological activities, particularly in anti-inflammatory and antiviral applications (Yan et al., 2021). Currently, an increasing number of active ingredients from traditional Chinese medicine are being identified and utilized across diverse sectors, including healthcare, functional foods, cosmetics, and flavors and fragrances, demonstrating substantial market potential and promising application prospects.

*Mosla chinensis* Maxim. cv. Jiangxiangru (McJXR), a cultivated variety of wild-type *M. chinensis* that belongs to the Lamiaceae (Labiatae) family, is a traditional Chinese medicine with good value for edible and medicinal purposes (Duan et al., 2024). It is mainly distributed in southern China, Vietnam, India and Japan (Li et al., 2014). The dried aerial parts of McJXR have heat-clearing, wetness-eliminating, analgesic, anti-vomiting, anti-inflammatory and antioxidant effects; they are thus widely used in the Chinese medicine industry (China Pharmacopoeia Committee CP, 2020) and serve as the main raw materials of many kinds of proprietary Chinese medicines. Furthermore, essential oil from dry aboveground parts of this plant is widely used in aromatherapy because of its unique aromatic properties, which can improve sleep quality, calm nerves and mitigate depression and anxiety (Wang et al., 2022). Owing to its distinctive aromatic odor, abundant volatile oil content, and diverse biological activities, McJXR exhibits considerable potential for application in the chemical industry, fragrance industry, and healthcare sector. Nevertheless, current studies on its chemical constituents predominantly focus on the isolation and identification of volatile oil components, with limited research addressing the non-volatile fractions.

Importantly, the distribution of these secondary metabolites exhibits clear tissue-specific characteristics. Chang et al. (2021) used ultra-performance liquid chromatography-quadrupole time-of-flight mass spectrometry (UPLC-Q-TOF/MS) to identify tissue-specific metabolites in different parts of *Platycodi Radix.* Their findings revealed that triterpene saponins were predominantly concentrated in the periderm, phloem and xylem, with the highest abundance observed in the phloem. Yuan et al. (2024) conducted a metabolomic analysis on the leaves, stems, and tubers of *Codonopsis convolvulacea* and found that the leaves were enriched in terpenoids, esters, and alcohols, whereas the stems exhibited high levels of terpenoids, heterocyclic compounds, and alkaloids, the tubers primarily accumulated carbohydrates such as sugars and starch. Wang et al. (2023) combined gas chromatography-mass spectrometry (GC-MS) and UHPLC-QTOF-MS to profile metabolites in the aerial parts of *Pogostemon cablin*. Their analysis revealed that the leaves contained the most diverse array of non-volatile compounds, while the inflorescences and lower leaves exhibited distinct and clearly tissue-specific biomarker profiles. However, to date, no studies have reported on the metabolic differences among various tissues of McJXR, which significantly hinders the comprehensive development and sustainable utilization of this valuable medicinal resource.

Plant metabolomics, a rapidly advancing field in post-genomic systems biology, aims to comprehensively identify and quantify small-molecule metabolites in plants (Yan et al., 2022). It enables the discovery of key metabolic markers linked to agronomic or medicinal traits and helps clarify associated pathways and regulatory networks. These insights support quality assessment, genetic improvement, and high-value use of medicinal plants (Xiao et al., 2022). LC-MS and GC-MS are the two core platforms in plant metabolomics, each with distinct strengths (Hong et al., 2016). They are complementary: LC-MS detects polar and thermally labile compounds, while GC-MS analyzes volatile and semi-volatile metabolites. Together, they reduce technical bias and provide broad metabolite coverage. In this study, a combined LC-MS and GC-MS analytical approach was employed to systematically profile the metabolite composition across different tissues of McJXR, conduct in-depth comparisons of their chemical profiles and variations, and identify tissue-specific candidate bioactive compounds. Furthermore, the antioxidant activity, total phenolic content (TPC), and total flavonoid content (TFC) across various tissues of McJXR were quantitatively assessed. Correlation analysis was conducted to investigate the potential pharmacologically active substance basis. The findings of this research will contribute to the efficient and sustainable utilization of McJXR, support the precise harvesting and development of its medicinal components, and provide a scientific basis for future studies on the biosynthesis and metabolic engineering of target bioactive compounds.

## 2 Materials and methods

### 2.1 Plant materials and experimental design

**Figure 1:**
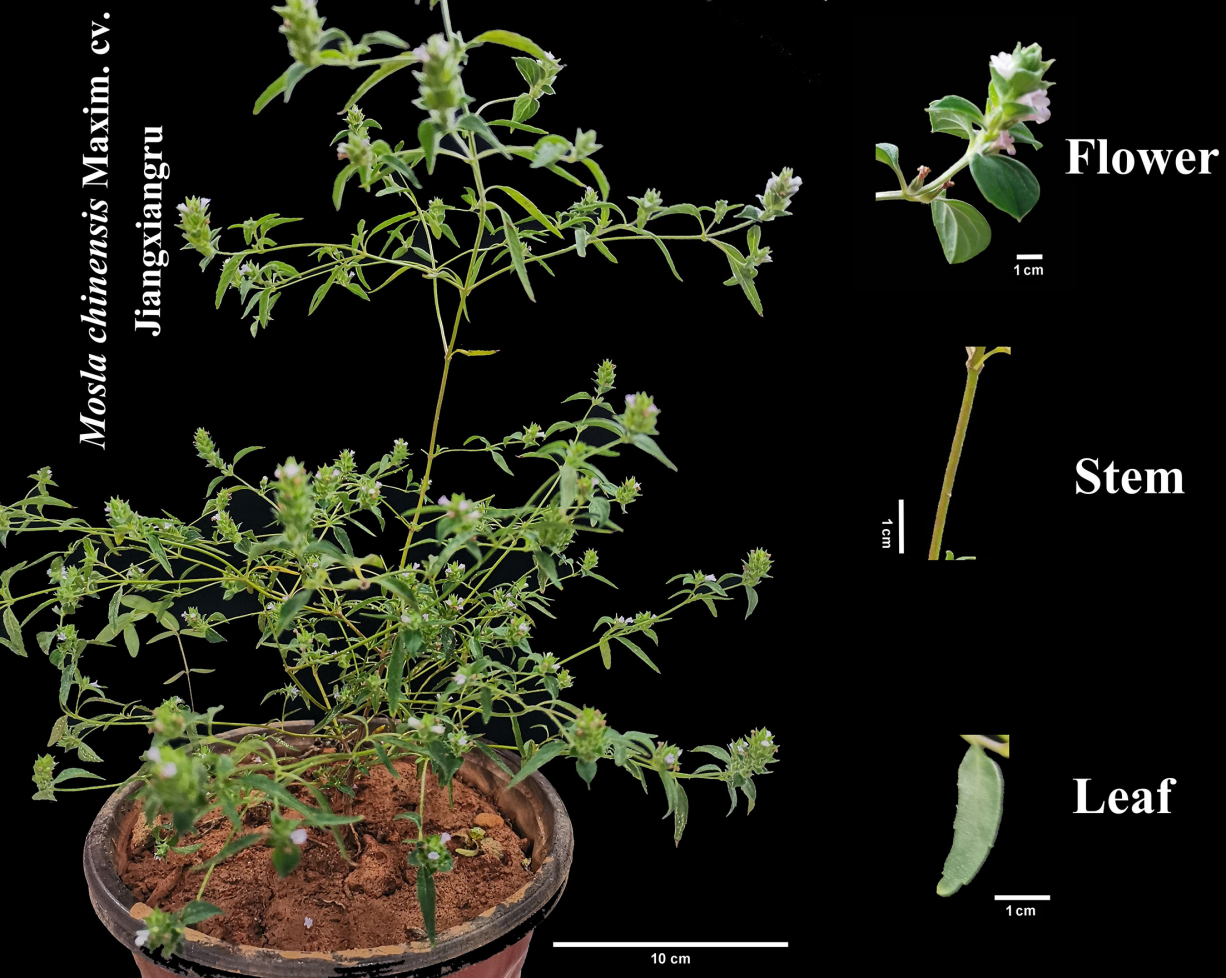
The phenotypic characteristics of *Mosla chinensis* Maxim. cv. Jiangxiangru plants and their leaf, stem and flower.

In this study, mature seeds of McJXR collected from its geo-authentic habitat in Fenyi County, Jiangxi Province, China, were used as propagation materials. Seedlings were uniformly cultivated in a greenhouse and subsequently grown into mature plants. At the flowering stage (3–4 months after sowing, **Figure 1**), healthy plants exhibiting consistent growth conditions and phenotypic characteristics were selected for sampling. Given that the traditional medicinal components of this plant are derived from the aboveground parts, three distinct tissues—flowers, leaves, and stems—were collected to comprehensively evaluate their metabolite profiles and associated biological activities. For each tissue type, three biological replicates were established, with each replicate comprising three independent plants, resulting in a total of nine samples. Immediately after collection, samples were flash-frozen in liquid nitrogen, thoroughly ground into fine powder, and stored at –80°C in an ultra-low temperature freezer for subsequent metabolomics analysis. Meanwhile, fresh samples of stems, leaves, and flowers were naturally dried and subsequently ground into powder using a laboratory grinder. One gram of powder from each tissue (flower, leaf, and stem) was added to 20 mL of 70% (v/v) (1:20) ethanol, followed by ultrasonic-assisted extraction for 30 minutes at 25 °C in an ultrasonic cleaner. The samples were then centrifuged at 4200 × g for 10 minutes to obtain the ethanol extract of different tissues, which were used for *in vitro* antioxidant activity assessment.

### 2.2 Extraction and LC-MS/MS analysis for non-volatile compounds

The extraction of non-volatile compounds from McJXR and subsequent LC-MS/MS analysis were performed in accordance with the methodology of Hua et al. (2023), with minor modifications. The detailed procedure is as follows: an appropriate quantity of McJXR leaf, stem, and flower tissues was accurately weighed and transferred into 2 mL centrifuge tubes, followed by the addition of 0.6 mL of 80% methanol solution containing 4 ppm 2-chlorophenylalanine as an internal standard (Chen et al., 2024). The mixture was vortexed for 30 seconds to ensure homogeneity, then ground at a frequency of 55 Hz for 60 seconds using a standard homogenizer. Subsequently, the samples were subjected to ultrasonication at room temperature for 15 minutes. Following this, the samples were centrifuged at 4°C and 12,000 rpm for 10 minutes. The supernatant was collected, filtered through a 0.22 μm microporous membrane, and transferred to an autosampler vial for LC-MS/MS analysis.

LC-MS/MS analysis was carried out using an ACQUITY UPLC^®^ HSS T3 column (2.1 × 100 mm, 1.8 μm, Waters) maintained at 40°C. The mobile phase was delivered at a flow rate of 0.3 mL/min, with an injection volume of 2 μL. The gradient elution profile was based on the method of Zelena et al. (2009), and the analysis was conducted in both positive and negative ionization modes. Mass spectrometric data were acquired using a Thermo Orbitrap Exploris 120 high-resolution mass spectrometer (Thermo Fisher Scientific, USA) coupled with an electrospray ionization (ESI) source operating in both positive and negative ion modes. The spray voltage was set to 3.50 kV in positive mode and −2.50 kV in negative mode. Sheath gas flow was maintained at 40 Arb, and auxiliary gas flow at 10 Arb. The capillary temperature was held at 325 °C. For the first-stage mass analysis, full-scan MS was performed at a resolution of 60,000 (m/z 100–1000) to ensure broad coverage of ion species. Tandem mass spectrometry (MS/MS) was carried out using HCD (higher-energy collisional dissociation) with a normalized collision energy of 30% and a resolution of 15,000. Data-dependent acquisition was employed to selectively fragment the four most intense precursor ions in each cycle. Dynamic exclusion was applied for 10s to minimize redundant fragmentation and enhance spectral quality, thereby improving overall detection sensitivity and data reliability (Want et al., 2013).

### 2.3 Metabolite extraction and GC-MS analysis

#### 2.3.1 Sample pretreatment

An appropriate quantity of frozen powder from the leaves, stems, and flowers of McJXR was accurately weighed and transferred into 2 mL centrifuge tubes. Subsequently, 0.5 mL of acetonitrile:isopropanol:water (2:2:1, v/v/v) mixed extraction solution was added. The samples were homogenized using a high-throughput tissue grinder at a frequency of 60 Hz for 90 seconds, with the grinding process repeated twice. Following homogenization, the samples were subjected to ultrasonication at room temperature for 5 minutes, after which an additional 0.5 mL of extraction solution was added, and ultrasonication was continued for another 5 minutes. The samples were then centrifuged at 4°C and 12,000 rpm for 5 minutes. A volume of 500 μL of the resulting supernatant was transferred to a new 2 mL centrifuge tube and completely dried under a vacuum concentrator. The dried residue was reconstituted in 80 μL of methoxyamine hydrochloride solution (20 mg/mL in pyridine), vortexed for 30 seconds to ensure homogeneity, and derivatized at 60°C for 60 minutes. Subsequently, 100 μL of BSTFA reagent containing 1% TMCS was added, and the mixture was further incubated at 70°C for 90 minutes. Upon completion of the reaction, the samples were centrifuged at 12,000 rpm for 5 minutes, and 90–100 μL of the supernatant was transferred to a glass insert for GC-MS analysis.

#### 2.3.2 Gas chromatography conditions

Chromatographic separation was performed using a Thermo Trace 1300 gas chromatograph (Thermo Fisher Scientific, USA) coupled with a Thermo ISQ 7000 single quadrupole mass spectrometer (Thermo Fisher Scientific, USA). The separation was carried out on an Rxi-5Sil MS capillary column (30 m × 0.25 mm × 0.25 μm). The injection volume was 1 μL, with a split ratio of 20:1. The injection port temperature was set at 280°C. High-purity helium was used as the carrier gas at a constant flow rate of 1.0 mL/min. The oven temperature program was as follows: initial temperature of 50°C held for 2 minutes, ramped at 5°C/min to 180°C, then increased at 10°C/min to 300°C, where it was held for 5 minutes. The transfer line temperature was maintained at 280°C.

#### 2.3.3 Mass spectrometry conditions

Mass spectrometric detection was carried out using an electron impact ionization (EI) source at an electron energy of 70 eV. The ion source temperature was set at 300°C. Full-scan mass spectra were acquired in SCAN mode over a mass range of 50–550 Da.

### 2.4 Preprocessing of metabolomics data and metabolite annotation

The preprocessing of raw metabolomics data and metabolite identification were primarily conducted in accordance with the methodologies established by Yan et al. (2023). Initially, raw mass spectrometry data were converted from instrument-specific formats into the standardized mzXML format using the MSConvert tool within the ProteoWizard software package (v3.0.8789) (Rasmussen et al., 2022). Subsequently, the XCMS package (v3.12.0) in R was employed for peak detection, filtering, and alignment, resulting in the generation of a quantitative feature matrix for metabolites (Navarro-Reig et al., 2015). The main parameters were set as follows: bw = 2, ppm = 15, peakwidth = c (5, 30), mzwid = 0.015, mzdiff = 0.01, method = centWave. The data matrix was obtained including the mass-to-charge ratio (m/z), and retention time (rt), and then the precursor molecules in positive and negative ion modes were generated. Subsequently, a robust LOESS-based signal correction method was applied to the data to eliminate systematic biases. Following standardization, only ion peaks exhibiting a relative standard deviation (RSD) below 30% in quality control samples (QC) were retained, ensuring accurate and reliable metabolite identification.

Following data standardization and scaling, machine learning analysis was performed using the R package Ropls (V1.22.0), encompassing unsupervised principal component analysis (PCA), supervised partial least squares discriminant analysis (PLS-DA), and orthogonal partial least squares discriminant analysis (OPLS-DA). Model overfitting was assessed through permutation testing. For the identification of significantly differentially metabolites (DMs), a combination of three statistical criteria was applied: P values derived from t-tests and variable importance in projection (VIP) scores from the OPLS-DA model. The threshold for significance was defined as P < 0.05 and VIP > 1.

GC–MS identification was primarily based on comparison with the NIST 2020 mass spectral library (similarity > 80%) and retention index consistency analysis, and is assigned to metabolite standard initiative (MSI) Level 2. Metabolite identification and pathway analysis of UHPLC-MS/MS were performed by PANOMIX Biomedical Tech Co., Ltd. (Suzhou, China). In the identification process, differential features were initially screened based on exact molecular mass with a mass accuracy of below 30 ppm. Subsequently, putative metabolite identities were confirmed through matching and annotation against multiple authoritative databases, including the Human Metabolome Database (HMDB, https://www.hmdb.ca), MassBank (https://massbank.eu/MassBank/), LipidMaps (https://www.lipidmaps.org), MzCloud (https://www.mzcloud.org), and PANOMIX’s in-house commercial database, thereby enabling accurate metabolite characterization. The molecular weight of metabolites was determined according to the m/z (mass-to-charge ratio) of parent ions in MS data. Molecular formula was predicted by ppm (parts per million) and adduct ion, and then matched with the database to realize MS identification of metabolites. At the same time, the MS/MS data from quantitative table of MS/MS data, were matched with the fragment ions and other information of each metabolite in the database, so as to realize the MS/MS identification of metabolites. In this study, metabolites were putatively identified to MSI level 2. Although non-targeted metabolomics carries a risk of false positives, this study has enhanced the credibility of metabolite annotations and their biological relevance through the application of stringent spectral matching criteria, cross-validation across multiple databases, and replication of associated metabolic patterns in an independent sample set. Finally, the DMs were subjected to Kyoto Encyclopedia of Genes and Genome (KEGG) pathway enrichment and topological analysis using the MetaboAnalyst platform, with the results visualized via the KEGG Mapper tool.

### 2.5 Determination of total phenolic content and total flavonoid content

The total flavonoid content was determined following the method of Li et al. (2015), employing the aluminum chloride colorimetric assay based on the formation of flavonoid-aluminum complexes to quantify the ethanol extracts from the three tissue types. This assay exhibits maximum absorbance at 510 nm, and a calibration curve was constructed using rutin as the reference standard. The results are expressed as milligrams of rutin equivalents per gram of dry weight extract (mg RE/g DW). Total polyphenol content was analyzed in accordance with the Chinese National Standard (GB/T 8313-2018) using the Folin-Ciocalteu method and expressed as milligrams of gallic acid equivalents per gram of dry weight extract (mg GAE/g DW) (Liang et al., 2022). All sample measurements were performed in triplicate.

### 2.6 Determination of antioxidant capacity

#### 2.6.1 Determination of DPPH radical scavenging activity

The experimental procedure was performed in accordance with the methodology described by Zhang et al. (2018). Specifically, a DPPH stock solution was prepared at a concentration of 0.4 mg/mL using methanol. A volume of 7 mL of this stock solution was transferred into a 100 mL volumetric flask and diluted to the mark with methanol to obtain the working solution. The initial absorbance at 517 nm was adjusted to fall within the range of 0.7–0.8. Subsequently, 2 mL of the working solution was combined with 0.5 mL of McJXR tissue sample extract. The mixture was vortexed thoroughly and incubated in the dark at room temperature for 30 minutes. Methanol was used as the blank control in place of the sample extract. The absorbance of each reaction mixture at 517 nm was measured using a UV-Vis spectrophotometer. A standard curve was constructed using Trolox (y = 126.953x + 0.023, R² = 0.998) to quantify DPPH radical scavenging activity.

#### 2.6.2 Determination of ABTS cation radical scavenging activity

The determination of ABTS radical scavenging activity was carried out in accordance with the method of Cheng et al. (2024), with appropriate experimental modifications. The detailed procedure is as follows: A 7.4 mM ABTS solution was mixed with a 2.6 mM potassium persulfate (K_2_S_2_O_8_) solution in equal volumes and allowed to react in the dark at room temperature for 12 hours to generate the ABTS^+^· radical stock solution. Prior to use, the stock solution was diluted approximately 50-fold with 10 mM phosphate buffer (pH 7.4), and the absorbance at 734 nm was adjusted to 0.70 ± 0.02 to prepare the ABTS^+^· working solution. A volume of 2.8 mL of the ABTS^+^· working solution was transferred into a test tube, followed by the addition of 0.2 mL of McJXR tissue sample extract. The mixture was vortexed thoroughly and incubated in the dark at room temperature for 6 minutes. The absorbance at 734 nm was subsequently measured using a UV-Vis spectrophotometer, with phosphate buffer used in place of the sample extract serving as the blank control. A standard curve was constructed using Trolox (y = 244.698x - 0.015, R² = 0.999) to quantify ABTS radical scavenging activity.

#### 2.6.3 Determination of ferric-reducing antioxidant power

The iron ion reducing capacity was determined in accordance with the method of Moghaddam et al. (2015), with appropriate modifications. The FRAP working solution was freshly prepared prior to use by mixing 0.1 mol/L acetate buffer (pH 3.6), 10 mM 2,4,6-tri(2-pyridyl)-S-triazine (TPTZ) solution (dissolved in 40 mM HCl), and 20 mM FeCl_3_ solution at a volume ratio of 2.5 mL: 2.5 mL: 2.5 mL. One milliliter of extract obtained from different tissues of McJXR was added to 2 mL of the FRAP working solution, followed by thorough mixing under light-protected conditions. The volume was then adjusted to 10 mL with double-distilled water, and the mixture was incubated in a water bath at 30 °C for 20 minutes. Absorbance was measured at 595 nm using a UV-Vis spectrophotometer. A standard curve was constructed using Trolox (y = 155.440x + 0.031, R² = 0.997) to quantify FRAP.

All antioxidant activity results were expressed in terms of milligrams of Trolox equivalent (TE) per gram of McJXR tissue powder in dry weight. All experiments were independently conducted in quintuplicate to ensure data reliability and reproducibility.

### 2.7 Statistical analysis

All experiments were independently replicated three times. Data are expressed as mean ± standard deviation (Mean ± SD, n = 3). Statistical differences among groups were analyzed using One-Way ANOVA with SPSS 24.0 software (SPSS Inc., Chicago, IL, USA). A P-value < 0.05 was considered statistically significant. When ANOVA indicated significant variation among groups, Tukey’s *post-hoc* test was applied for pairwise multiple comparisons. Furthermore, the potential correlations between metabolite contents and the total antioxidant capacity across different tissues of McJXR were evaluated using Pearson correlation analysis in SPSS 24.0. The level of statistical significance was set at P < 0.05 or P < 0.01.

## 3 Results

### 3.1 GC-MS analysis of compounds in different tissues of McJXR

**Figure 2:**
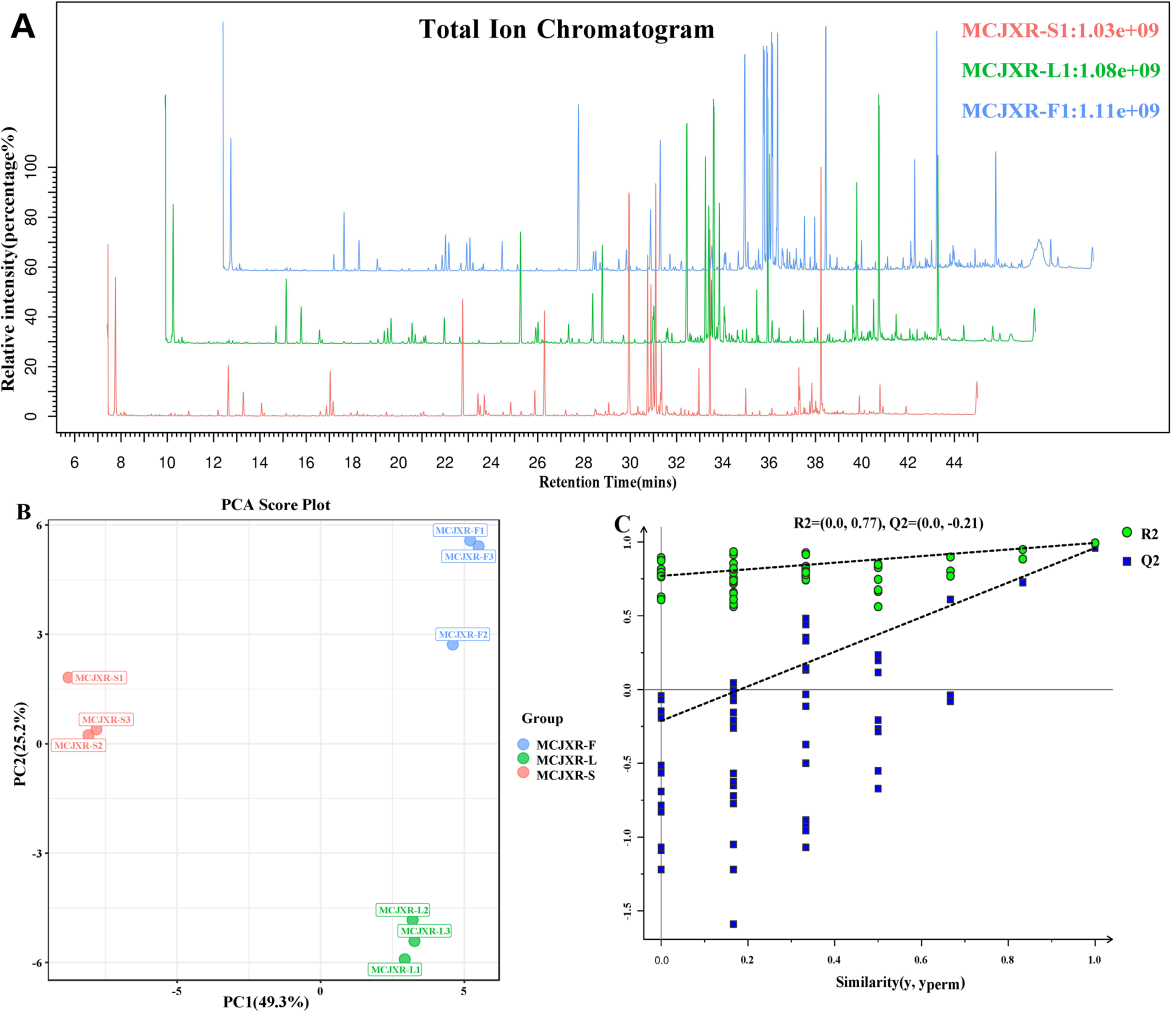
The results of a multivariate analysis of the volatile components in three types of *Mosla chinensis* Maxim. cv. Jiangxiangru tissue samples. **(A)** Typical total ion chromatogram of representative samples. **(B)** PCA score plots. **(C)** OPLS-DA permutation test plots.

Plants of the Lamiaceae family are widely recognized for their abundant and diverse aromatic oils, and are typically characterized by distinct and potent fragrances. In this study, GC-MS was employed to systematically profile the compounds in the stems, leaves, and flowers of McJXR (**Figure 2A**). PCA score plot revealed that samples of the same tissue type clustered closely, indicating good methodological stability and high reproducibility of the experimental results. Meanwhile, a clear separation between different tissue types was observed, suggesting substantial differences in their metabolite profiles (**Figure 2B**). To further explore the metabolic distinctions among tissues, orthogonal partial least squares discriminant analysis (OPLS-DA) was conducted to construct a discriminatory model. The model exhibited R^2^X, R^2^Y, and Q^2^values of 0.743, 0.994, and 0.960, respectively, demonstrating strong explanatory capacity and robust predictive accuracy (**Figure 2C**).

**Figure 3:**
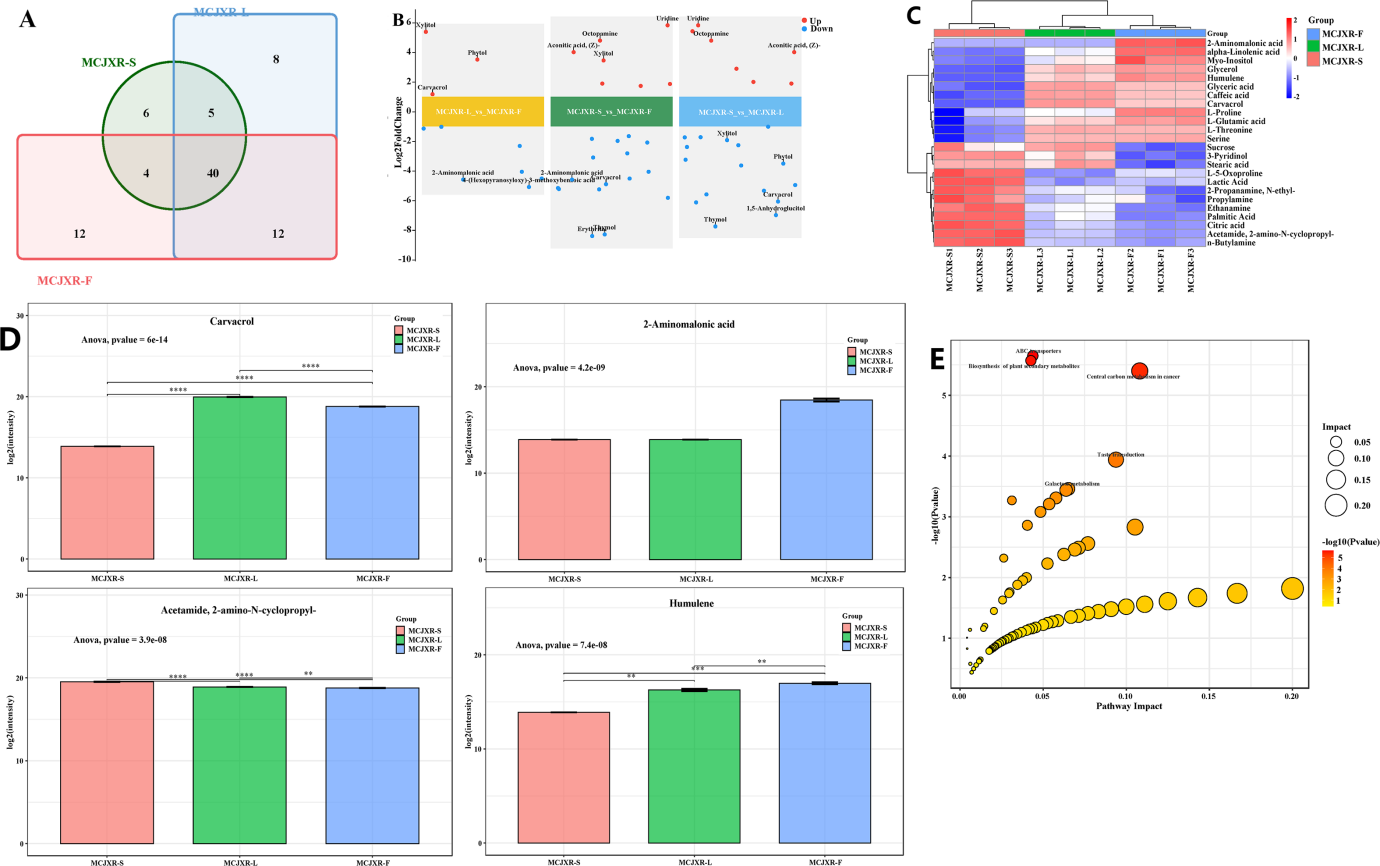
Identification and differential analysis of volatile compounds among three tissues of *Mosla chinensis* Maxim. cv. Jiangxiangrua. **(A)** Venn plot for the number of volatile compounds among three tissues. **(B)** Multiple differential volcano plots of differential volatile substances across distinct comparison groups. **(C)** Heatmap of differential volatile substances among three tissues. **(D)** Barplot of characteristic volatile substances among three tissues. **(E)** Bubble chart of the KEGG impact factors for different metabolic pathways.

A total of 87 compounds were identified across the three tissue types, with 55, 65, and 68 compounds detected in stems, leaves, and flowers, respectively (**Figure 3A**; **Supplementary Tables S1**-**S3**). Among these, 40 compounds were common to all three tissues, while 8, 6, and 12 compounds were uniquely present in leaves, stems, and flowers, respectively. Compounds shared between two tissues but not all three included Benzonitrile, 2-chloro-, Benzoic acid, Thymol, and Humulene, which were exclusively detected in leaves and flowers; Galactaric acid, L-Valine, and Dianthoside, which were found only in stems and flowers; and Xylitol and L-Isoleucine, which were restricted to stems and leaves. In addition, the leaves contain eight unique compounds, including 2,6-Di-tert-butyl-4-mercaptophenol, S-acetyl-, Phytol, Adonitol, etc. The flowers contain twelve unique compounds, such as beta-Alanine, Putrescine, Hexanoic acid, p-Cymene, etc. The stems contain six unique compounds, including Glycolic acid, Uridine, L-Rhamnose, etc. The tissue-specific distribution patterns of these compounds provide crucial insights into the biochemical basis of aroma formation in McJXR and inform the selection of its medicinal tissues.

Based on the screening criteria of p-value ≤ 0.05 and VIP ≥ 1, a total of 42 DMs (21 up-regulated and 21 down-regulated) were identified in the comparison of MCJXR-L vs MCJXR-F, 43 (20 up-regulated and 23 down-regulated) in MCJXR-S vs MCJXR-F, and 40 (19 up-regulated and 21 down-regulated) in MCJXR-S vs MCJXR-L (**Figure 3B**). Furthermore, 24 DMs were detected through cross-comparison among the three tissue types (**Figure 3C**). The four most significantly differentiated compounds were Carvacrol, 2-Aminomalonic acid, Acetamide, 2-amino-N-cyclopropyl-, and Humulene (**Figure 3D**). KEGG pathway enrichment analysis revealed that these DMs were predominantly enriched in the ABC transporter (map02010), plant secondary metabolite biosynthesis (map01060), taste transduction (map04742), and galactose metabolism (map00052) pathways (**Figure 3E**).

### 3.2 Characterization of non-volatile metabolites across different tissues of McJXR

#### 3.2.1 Comprehensive analysis

**Figure 4:**
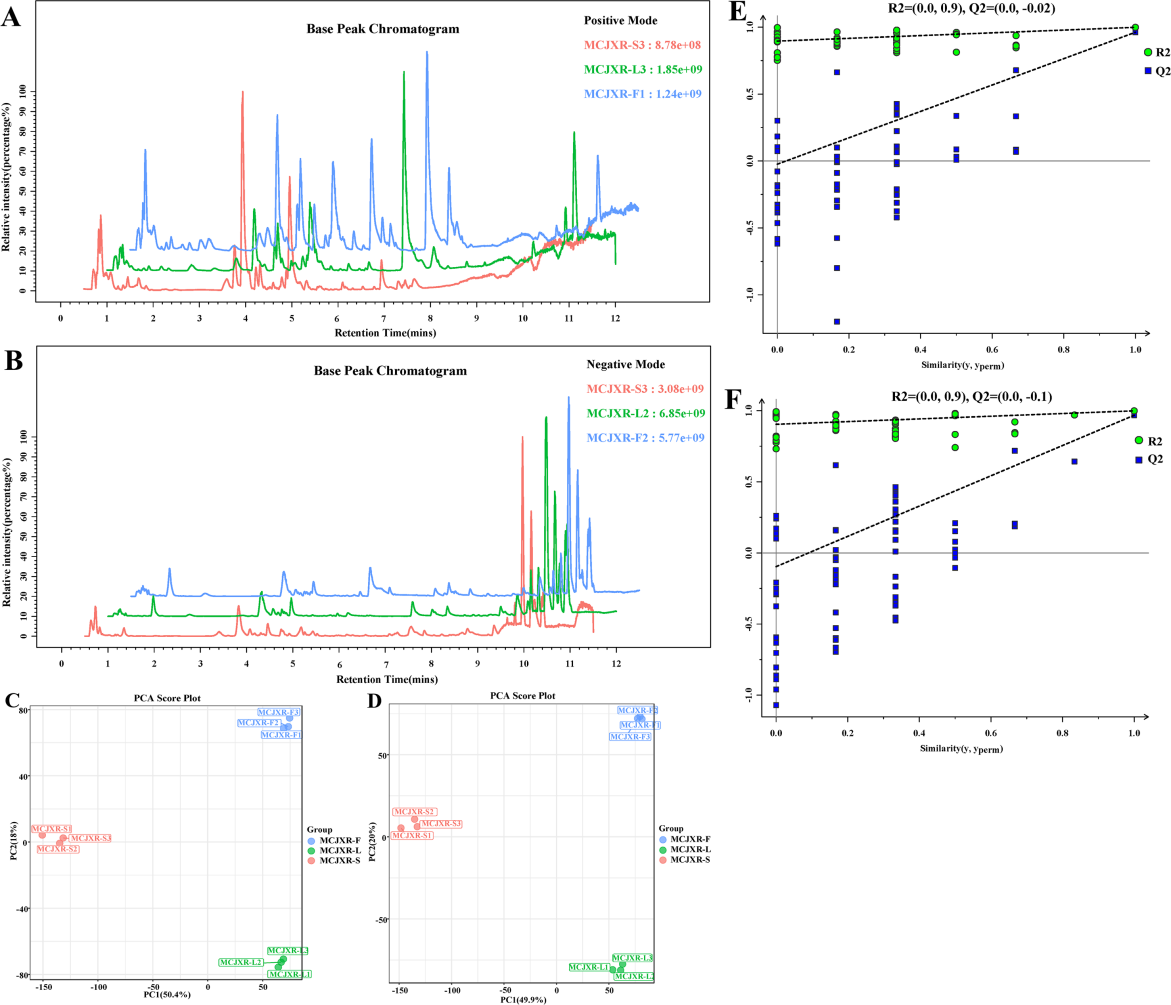
The multivariate statistical analysis of non-volatile compounds in different tissue samples of *Mosla chinensis* Maxim. cv. Jiangxiangrua. **(A)** Base peak chromatogram (BPC) in positive ion mode. **(B)** BPC in negative ion mode. **(C)** Principal component analysis (PCA) score plot in positive ion mode. **(D)** PCA score plot in negative ion mode. **(E)** OPLS-DA permutation test plot in positive ion mode. **(F)** OPLS-DA permutation test plot in negative ion mode.

To systematically assess the compositional differences of non-volatile metabolites across different tissues of McJXR, a non-targeted metabolomics approach was performed on stem, leaf, and flower samples using LC-MS/MS-based detection technology. A total of 21,636 ion features were detected in positive ion mode (**Figure 4A**), and 21,880 ion features were identified in negative ion mode (**Figure 4B**; **Supplementary Tables S4**, **S5**). Base peak chromatograms (BPCs) revealed that all samples exhibited high signal response intensities, well-defined chromatographic peaks, and substantial peak capacity, indicating effective chromatographic separation and high data quality, thereby supporting reliable metabolite identification and quantitative analysis.

PCA revealed significant differences in the composition of non-volatile metabolites among various tissue samples of McJXR (**Figures 4C, D**). Under the positive ion mode, the variance contribution rates of the PC1 and PC2 were 50.4% and 18.0%, respectively; under the negative ion mode, these values were 49.9% and 20.0%, respectively. PCA score plots demonstrated that PC1 primarily captured inter-group variation, whereas PC2 mainly represented intra-group variation. To more precisely characterize metabolic differences across tissues and identify discriminative metabolites, an OPLS-DA model was constructed. The results of the OPLS-DA permutation tests under both positive and negative ion modes are presented in **Figures 4E, F**, respectively. Model parameters under the positive ion mode were R^2^X = 0.684, R^2^Y = 0.999, and Q^2^ = 0.962, and under the negative ion mode were R^2^X = 0.699, R^2^Y = 0.999, and Q^2^ = 0.968. These metrics collectively indicate that the models exhibit strong explanatory capacity, robust predictive performance, and high stability, thereby confirming the significant compositional differences in non-volatile metabolites among different tissues.

#### 3.2.2 Differences in nonvolatile metabolites

**Figure 5:**
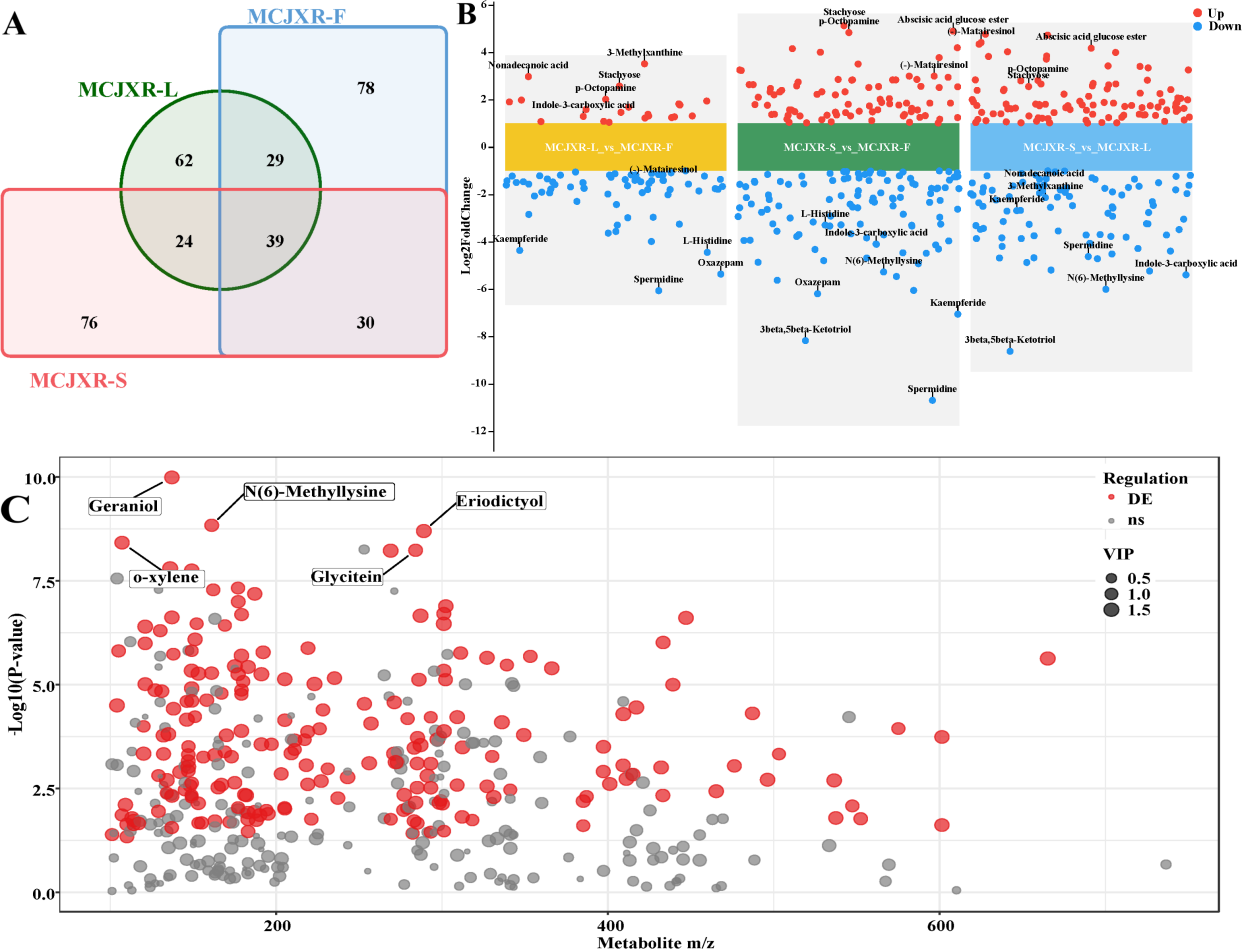
Comparative analysis of non-volatile metabolite composition in stem, leaf, and flower tissues of McJXR. **(A)** Venn diagram showing tissue-specific and shared non-volatile metabolites across different tissues. **(B)** Volcano plot depicting differentially non-volatile metabolites among pairwise tissue comparisons. **(C)** Scatter plot illustrating the relationship between VIP values and p-values of significantly differential metabolites from the cross-comparison of three tissue groups.

The identification and analysis of non-volatile metabolites in the stem, leaf, and flower tissues of McJXR revealed that the flower contained the highest number of metabolite types (8704: 8528 Level 1, 176 Level 2), followed by the stem (8418: 8249 Level 1, 169 Level 2), with the leaf containing the fewest (8839: 8685 Level 1, 154 Level 2) (**Supplementary Tables S6**-**S8**). Venn diagram analysis demonstrated the presence of 62, 78, and 76 tissue-specific metabolites in the leaf, flower, and stem, respectively, along with 39 commonly shared metabolites across all three tissues (**Figure 5A**). Further investigation confirmed that metabolite accumulation exhibited significant tissue-specific patterns. Metabolites enriched in both the leaf and flower primarily included alcohols and polyols, isoflav-2-enes, dicarboxylic acids and their derivatives, as well as short-chain keto acids and derivatives. In contrast, the leaf and stem showed higher enrichment of sesquiterpenoids, phenols and their derivatives, and androstane steroids. The stem and flower were characterized by elevated levels of amino acids, peptides, and analogues; carbohydrates and carbohydrate conjugates; purines and purine derivatives; and hydroxycinnamic acids and their derivatives. Additionally, the stem exhibited specific enrichment of carbonyl compounds, benzenediols, and pyridinecarboxylic acids and derivatives; the flower showed significant accumulation of monoterpenoids, 1-hydroxy-2-unsubstituted benzenoids, and flavones; whereas the leaf was predominantly enriched in amines, tetraterpenoids, and O-methylated isoflavonoids. In summary, the distinct distribution patterns of metabolites across different tissues of McJXR indicate that their biosynthesis and accumulation are highly tissue-specific, likely reflecting their roles in tissue-specific physiological functions and metabolic regulation.

Based on the screening criteria of p-value < 0.05 and VIP > 1.0, a substantial number of significantly DMs were identified through pairwise comparisons of stem, leaf, and flower tissues. Specifically, 271 DMs were detected between stem and leaf, including 149 up-regulated and 122 down-regulated; 267 DMs were observed between stem and flower (122 up-regulated and 145 down-regulated); and 192 DMs were identified between leaf and flower (65 up-regulated and 127 down-regulated) (**Figure 5B**). In the MCJXR-L vs. MCJXR-F, the top five metabolites ranked by VIP values were Spermidine, 3-[(1-Carboxyvinyl)oxy]benzoate, Tropate, L-Lysine, and L-Proline. In the MCJXR-S vs. MCJXR-F, the top five VIP-ranked metabolites were Kaempferide, Geraniol, Glycitein, Flavonol 7-O-beta-D-glucoside, and N(6)-Methyllysine. In the MCJXR-S vs. MCJXR-L, the top five VIP-ranked metabolites included Geraniol, (3S,5S)-3,5-Diaminohexanoate, N(6)-Methyllysine, Glycitein, and o-Xylene. A cross-comparison of the three groups revealed a total of 212 significantly DMs (**Figure 5C**). Among these, the five compounds with the lowest p-values (i.e., highest statistical significance) were Geraniol, N(6)-Methyllysine, Eriodictyol, o-Xylene, and Glycitein. Based on VIP scores, the top five metabolites were Luteolin, p-Octopamine, Apiole, Fisetin, and Kaempferide. These findings indicate that the accumulation of metabolites varies significantly across different tissues of McJXR, demonstrating clear tissue-specific metabolic characteristics. This pattern suggests a potential association with tissue-specific biological functions and regulatory mechanisms.

#### 3.2.3 KEGG pathway enrichment analysis for differential metabolites

**Figure 6:**
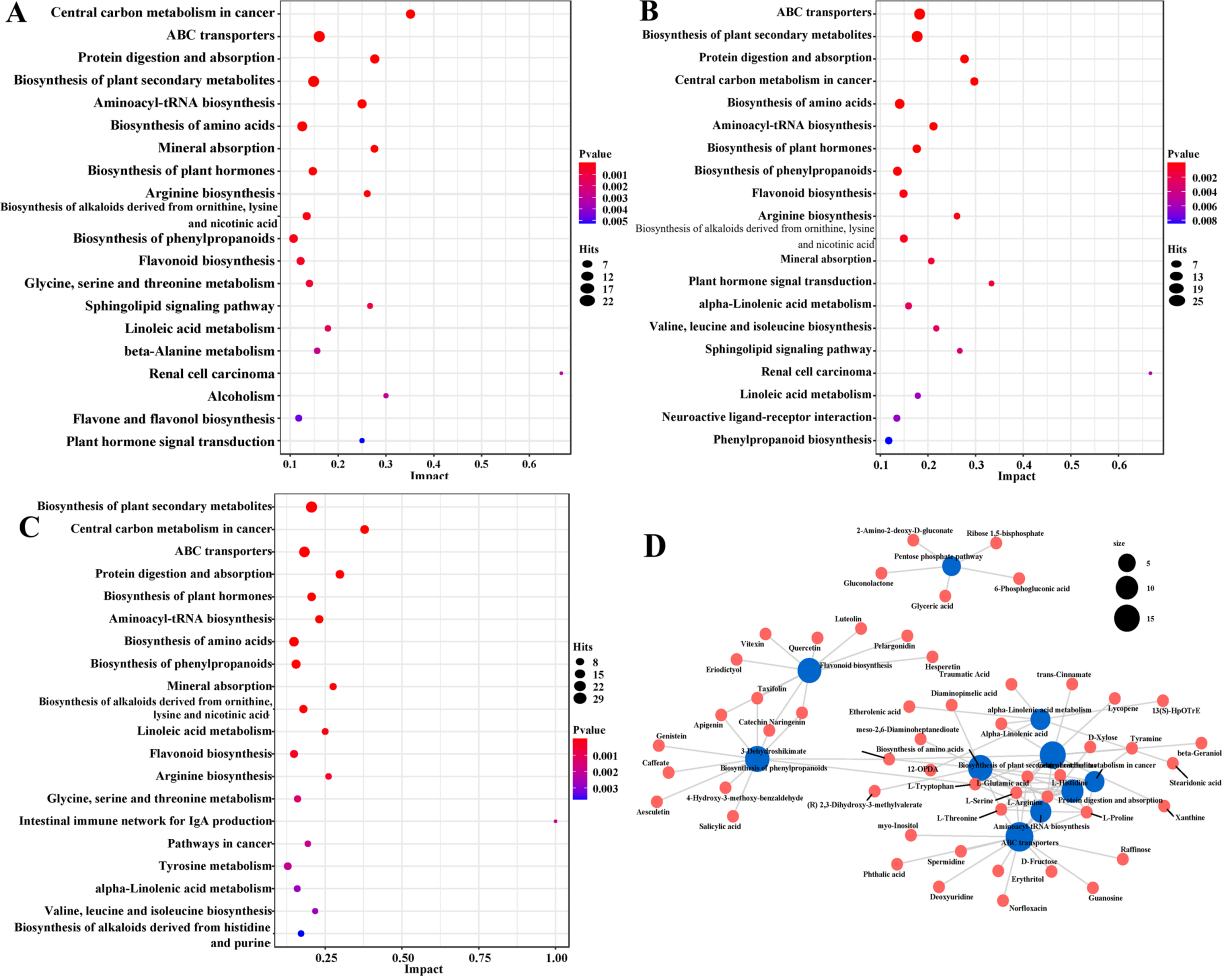
KEGG enrichment maps and metabolite-metabolic pathway interaction network of differential metabolites for three comparison groups (MCJXR-L vs. MCJXR-F, MCJXR-S vs. MCJXR-F, MCJXR-S vs. MCJXR-L). **(A–C)** KEGG enrichment maps of differential metabolites in different pairwise comparisons: **(A)** MCJXR-L vs. MCJXR-F; **(B)** MCJXR-S vs. MCJXR-F; **(C)** MCJXR-S vs. MCJXR-L. **(D)** The metabolite-metabolic pathway interaction network of differential metabolites in MCJXR-S vs. MCJXR-L vs. MCJXR-F.

To further elucidate the potential metabolic pathways and biological functions of differential metabolites in the leaves, stems, and flowers of McJXR, KEGG enrichment analysis was performed on the significantly DMs. The results revealed that, across different tissue comparison groups, these metabolites were significantly enriched in several key metabolic pathways: ABC transporters (map02010), Protein digestion and absorption (map04974), Biosynthesis of plant secondary metabolites (map01060), Aminoacyl-tRNA biosynthesis (map00970), Biosynthesis of amino acids (map01230), and Biosynthesis of plant hormones (map01070) (**Figures 6A–C**). Notably, in the three-way comparison (MCJXR-S vs. MCJXR-L vs. MCJXR-F), the commonly DMs were significantly enriched in the following pathways: ABC transporters (map02010), Protein digestion and absorption (map04974), Flavonoid biosynthesis (map00941), Biosynthesis of phenylpropanoids (map01061), and Biosynthesis of plant secondary metabolites (map01060) (**Figure 6D**).

### 3.3 Differences in TPC, TFC, and antioxidant activity in different tissues of McJXR

**Figure 7:**
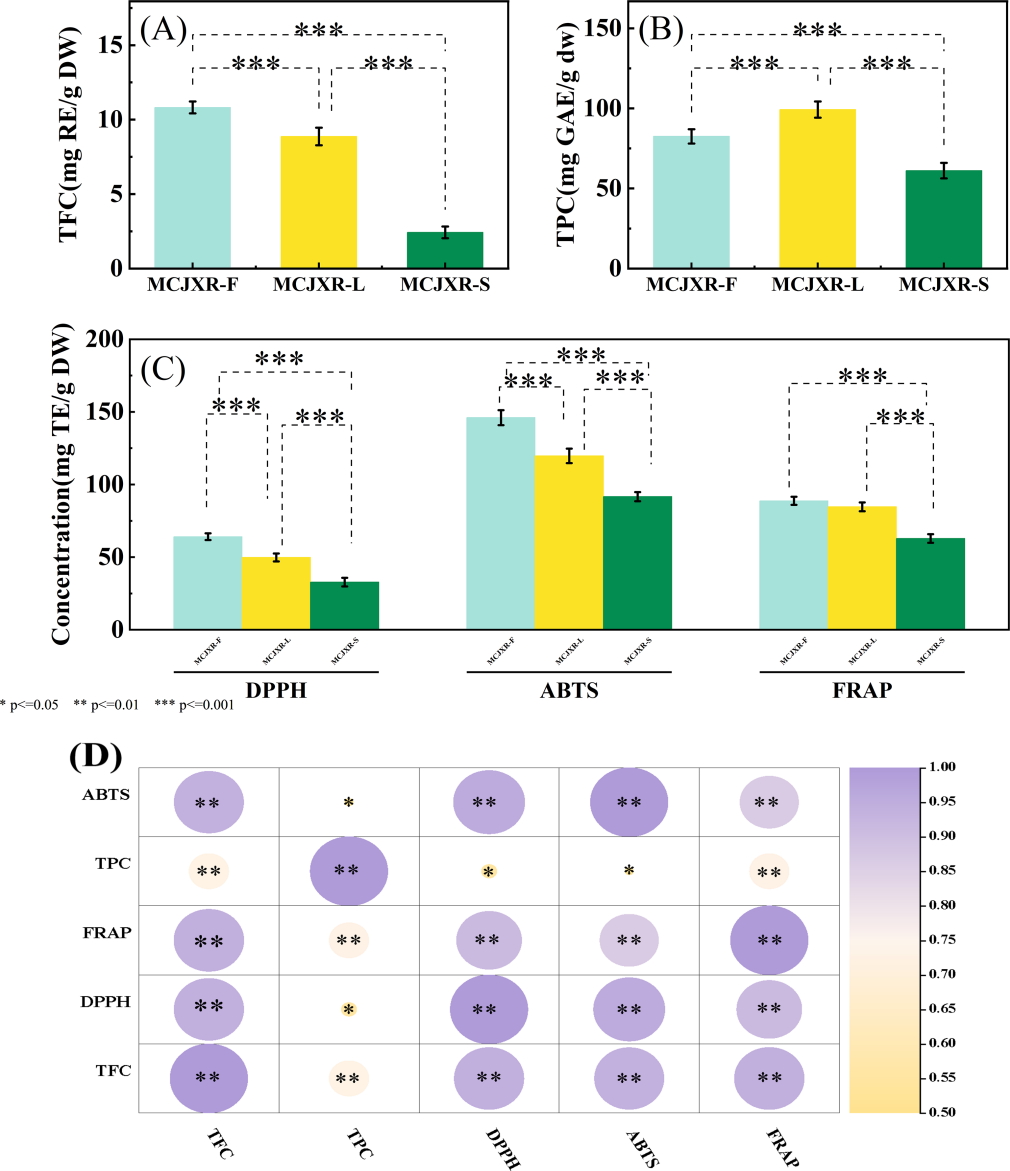
The TPC, TFC, and antioxidant capacity of different tissues of McJXR. **(A)** TFC of different tissues. **(B)** TPC of different tissues. **(C)** Antioxidant capacity of different tissues. **(D)** The heat map of correlation coefficients of TPC, TFC, and three antioxidant activity. Statistical significance of group differences was assessed using the Tukey’s Honestly Significant Difference test; *, **, *** denote P ≤ 0.05, P ≤ 0.01 and P ≤ 0.001, respectively.

McJXR is a traditional Chinese medicine rich in polyphenols and flavonoids, exhibiting excellent antioxidant activity by effectively scavenging free radicals in the body and thereby mitigating oxidative stress-induced damage. In this study, the TPC and TFC in the flowers, leaves, and stems of McJXR were quantified, and the antioxidant capacities of different plant tissues were systematically compared using three *in vitro* antioxidant assays: DPPH, FRAP, and ABTS. The results revealed significant variations in the levels of bioactive constituents across different tissues. As shown in **Figure 7A**, the TFC was highest in the flowers (10.82 mg RE/g DW), followed by the leaves, with the lowest content detected in the stems (2.43 mg RE/g DW). The distribution pattern of TPC differed slightly (**Figure 7B**), with the highest content found in the leaves (99.25 mg GAE/g DW), followed by the flowers, and the lowest in the stems (61.17 mg GAE/g DW). Antioxidant assay results (**Figure 7C**) demonstrated a consistent activity trend across all three methods: MCJXR-F > MCJXR-L > MCJXR-S. However, variations in antioxidant activity were observed for the same sample across different assays, likely due to differences in the underlying reaction mechanisms of the DPPH, ABTS, and FRAP methods.

Overall, the TFC, TPC, and all three antioxidant indices were significantly higher in the flowers and leaves than in the stems, indicating stronger antioxidant capacity in these tissues, which is closely associated with their higher contents of flavonoids and polyphenols. Therefore, a correlation analysis was conducted between TPC, TFC, and antioxidant activity. As illustrated in **Figure 7D**, both TFC and TPC showed significant positive correlations with all three antioxidant activities (r > 0.5000, p < 0.05), suggesting that polyphenols and flavonoids play a key role in free radical scavenging. Moreover, TFC and TPC were highly significantly correlated (r = 0.7426, p < 0.01), indicating that flavonoids may represent the major polyphenolic constituents in McJXR. Furthermore, TFC exhibited highly significant positive correlations with ABTS (r = 0.9443, p < 0.01), FRAP (r = 0.9468, p < 0.01), and DPPH (r = 0.9459, p < 0.01) antioxidant capacities, reinforcing the conclusion that flavonoids are the primary bioactive components responsible for the antioxidant and radical-scavenging effects of McJXR.

### 3.4 Correlation between metabolites and antioxidant activities

**Figure 8:**
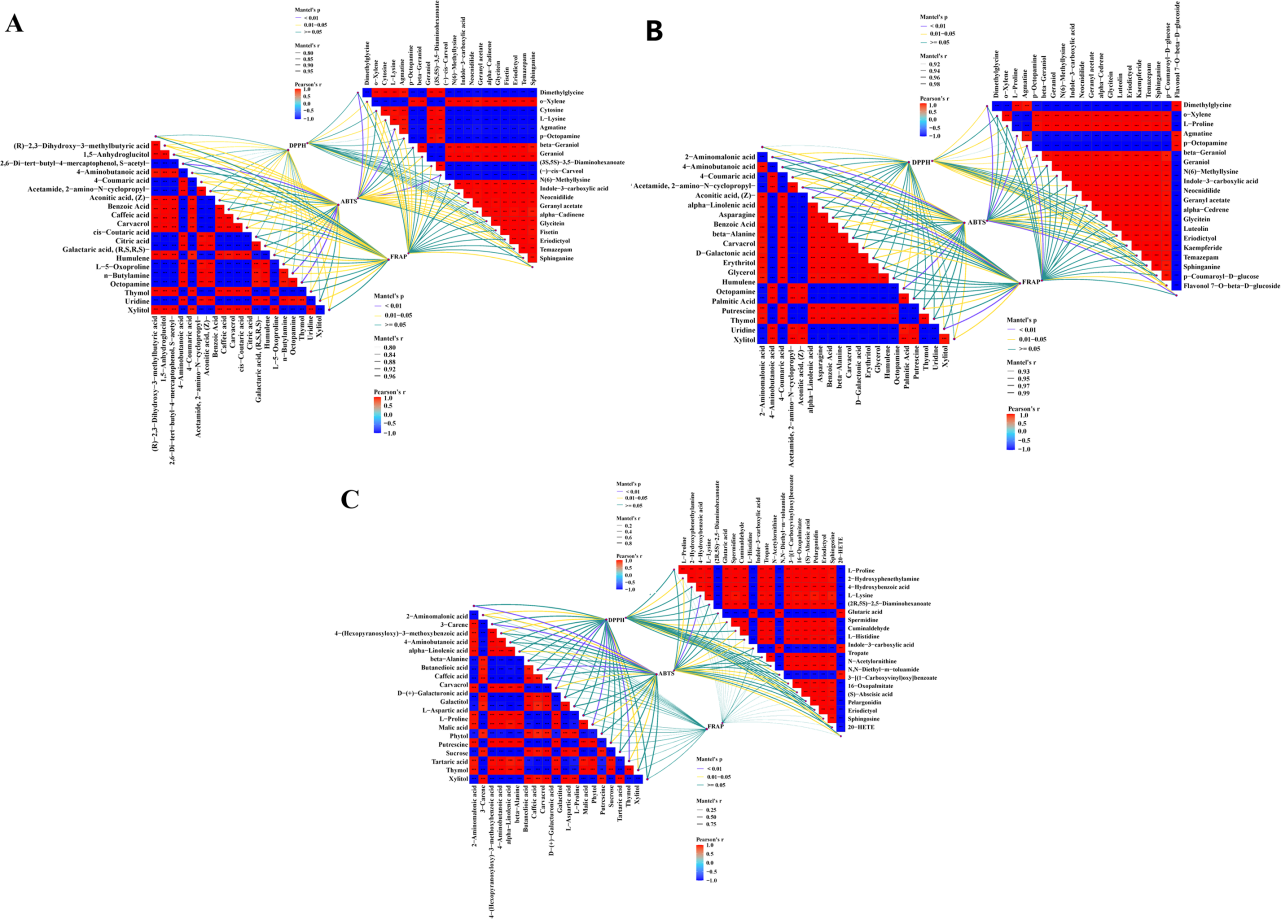
Mantel test results for significantly different metabolites (top 20 VIP values) and antioxidant indicators (ABTS, DPPH, FRAP) across different tissues of McJXR. **(A)** MCJXR-S vs. MCJXR-L. **(B)** MCJXR-S vs. MCJXR-F. **(C)** MCJXR-L vs. MCJXR-F. The lower-left quadrant displays significantly different volatile metabolites, while the upper-right quadrant displays significantly different non-volatile metabolites.

To identify potential antioxidant active substances in McJXR, this study conducted Mantel tests based on significantly different metabolites across various tissues (top 20 VIP values) and antioxidant indicators (ABTS, DPPH, FRAP), systematically analyzing the associations of metabolites and antioxidant activity in three tissue comparisons: MCJXR-S vs. MCJXR-L, MCJXR-S vs. MCJXR-F, and MCJXR-L vs. MCJXR-F. In the MCJXR-S vs. MCJXR-L (**Figure 8A**), ABTS radical scavenging activity showed significant positive correlations with metabolites including carvacrol, thymol, humulene, xylitol, octopamine, and 4-aminobutyric acid (r > 0.90, p < 0.05). DPPH radical scavenging activity was significantly correlated with thymol, carvacrol, and xylitol (r > 0.80, p < 0.05), while FRAP reducing capacity was closely associated with metabolites such as caffeic acid and 4-coumaric acid (r > 0.90, p < 0.05). Among non-volatile metabolites, ABTS was significantly correlated with (-)-cis-carveol, agmatine, dimethylglycine, and L-lysine; DPPH correlated with sphinganine, L-lysine, and o-xylene; and FRAP was significantly correlated with sphinganine, p-octopamine, and cytosine. In the MCJXR-S vs. MCJXR-F (**Figure 8B**), ABTS was significantly correlated with 4-aminobutyric acid, octopamine, and xylitol; DPPH correlated with 4-aminobutyric acid and octopamine; and FRAP was significantly correlated with xylitol. Among non-volatile metabolites, ABTS was significantly correlated with dimethylglycine, spermidine, Flavonol 7-O-beta-D-glucoside, and luteolin; DPPH correlated with Flavonol 7-O-beta-D-glucoside, L-proline, and p-octopamine; and FRAP was significantly correlated with p-octopamine, p-coumaroyl-D-glucose, and spermidine. In the MCJXR-L vs. MCJXR-F (**Figure 8C**), metabolites significantly correlated with ABTS included caffeic acid, tartaric acid, and putrescine; those correlated with DPPH included malic acid, tartaric acid, 4-aminobutyric acid, and thymol. No volatile metabolites were found to be significantly correlated with FRAP. Among non-volatile metabolites, ABTS was significantly correlated with L-lysine, 2-hydroxyphenethylamine, and 20-hydroxyeicosatetraenoic acid (20-HETE); DPPH correlated with indole-3-carboxylic acid and N,N-diethyl-m-toluamide; no significant correlations with FRAP were observed.

In summary, through systematic analysis of the correlations between differential metabolites and antioxidant activities across three tissue comparisons using Mantel tests, this study identified multiple potential antioxidant active substances significantly associated with the antioxidant capacity of McJXR. Notably, octopamine, 4-aminobutyric acid, xylitol, thymol, carvacrol, humulene, and coumaric acid exhibited stable and highly significant positive correlations with antioxidant indicators such as ABTS and DPPH across multiple comparisons, suggesting that these compounds may represent potential antioxidant-related metabolites associated with the antioxidant effects of McJXR. Furthermore, certain flavonoids, amino acids, and phenolic acids were found to be significantly associated with antioxidant activity in specific tissues, highlighting the chemical diversity and tissue-specificity of the antioxidant properties in McJXR. These findings provide a valuable foundation for elucidating the chemical basis of antioxidant activity and for further exploration of key functional components in this species.

## 4 Discussion

### 4.1 Tissue-specific metabolite accumulation patterns in McJXR and their ecological-physiological implications

To elucidate the metabolic characteristics of different tissues in McJXR and support their precise utilization, we conducted a comprehensive metabolite profiling of stems, leaves, and flowers using GC–MS and LC–MS/MS. A distinct tissue-specific accumulation pattern was observed, consistent with the established principle of spatiotemporal specificity in plant secondary metabolism (Yuan et al., 2024; Liu et al., 2023), yet featuring unique metabolic signatures characteristic of McJXR.

GC–MS analysis identified 87 metabolites in total, with the highest number detected in flowers (68), followed by leaves (65) and stems (55). Each tissue contained exclusive marker compounds: flowers harbored 12 unique constituents (e.g., p-cymene, putrescine), leaves contained 8 (e.g., 2,6-di-tert-butyl-4-mercaptophenol, phytol), and stems featured 6 (e.g., glycolic acid, uridine). LC–MS/MS (MSI level 2) identified 426 compounds, with flowers exhibiting the greatest chemical diversity (176), followed by stems (169) and leaves (154). Specifically, flowers were enriched in flavonoids (e.g., luteolin, apigenin) and monoterpenoids; leaves in amines, tetraterpenoids, and O-methylated isoflavones; and stems in carbonyl compounds, phenolic acids, and pyridine carboxylic acid derivatives. These tissue-specific metabolic profiles reflect the functional specialization of McJXR organs.

As reproductive structures, McJXR flowers accumulate volatile monoterpenes (e.g., p-cymene) and flavonoids—traits likely adaptive for pollinator attraction (Li et al., 2025) and protection against UV radiation and pathogens (Shomali et al., 2022), both critical for reproductive success. Leaves, serving as primary photosynthetic organs, accumulate amines and phenolics, aligning with their role in defense against biotic and abiotic stresses such as herbivory, high light intensity, and drought (Gago et al., 2016). The high polyphenol and flavonoid content in leaves accounts for their strong ABTS radical scavenging activity, a direct physiological adaptation to photooxidative stress. Stems, functioning as structural supports and transport conduits, are enriched in metabolites associated with mechanical reinforcement (e.g., lignin precursors) and systemic signaling in defense responses, as evidenced by their distinct accumulation of phenolic acids and carbonyl compounds. The spatiotemporal regulation of gene expression (Wang et al., 2023) is the primary mechanism underlying these tissue-specific metabolic patterns, which our data directly corroborate in McJXR.

### 4.2 Key metabolic pathways regulating secondary metabolism in McJXR based on differential metabolite enrichment

KEGG enrichment analysis of DMs revealed core pathways governing the biosynthesis of bioactive compounds in McJXR, including ABC transporters, biosynthesis of plant secondary metabolites, protein digestion and absorption, plant hormone biosynthesis, and amino acid biosynthesis. These pathways were consistently enriched across multiple tissue comparisons, underscoring their central regulatory roles in metabolic organization. The ABC transporter pathway was significantly enriched, which is directly relevant to the observed tissue-specific accumulation of active compounds. ABC transporters mediate the translocation of secondary metabolites—such as flavonoids and alkaloids—from sites of synthesis to vacuoles or apoplastic spaces (Yazaki, 2006; Gani et al., 2021). In McJXR, the elevated accumulation of thymol, carvacrol, and flavonoids in flowers and leaves may depend not only on biosynthetic capacity but also on the tissue-specific expression of ABC transporters. This interpretation is supported by the consistent enrichment of this pathway in flower vs. stem and leaf vs. stem comparisons, indicating its role in the compartmentalization and spatial distribution of key metabolites.

Flavonoid biosynthesis and phenylpropanoid biosynthesis were also significantly enriched, directly accounting for the high levels of flavonoids (e.g., luteolin, apigenin), phenolic acids (e.g., caffeic acid), and their derivatives in McJXR flowers and leaves. Phenylpropanoid metabolism plays a central role in plant antioxidant defense (Dong and Lin, 2021), and the enhanced activity of this pathway in flowers and leaves—consistent with our metabolite profiles—correlates with their superior DPPH, ABTS, and FRAP radical scavenging activities. The tissue-specific expression of key structural genes (*PAL, C4H, 4CL, CHS*) and regulatory transcription factors (MYB, bHLH) in these pathways (Ma et al., 2024; Yang et al., 2024) likely underpins metabolic divergence in McJXR, a mechanism indirectly validated by our pathway enrichment results.

Enrichment of amino acid biosynthesis pathways suggests tissue-specific reprogramming of nitrogen metabolism in McJXR. Amino acids serve as precursors for various secondary metabolites, including alkaloids and glucosinolates, and shifts in their metabolic flux can reshape downstream metabolic networks (Yang et al., 2020). Furthermore, the enrichment of central carbon metabolism pathways—glycolysis, the TCA cycle, and the pentose phosphate pathway—indicates an increased supply of biosynthetic precursors (e.g., PEP, E4P) and reducing power (NADPH) in flowers and leaves, supporting the active biosynthesis of phenylpropanoids and flavonoids in these tissues (Timm and Arrivault, 2021). Collectively, these interconnected pathways constitute a coordinated regulatory module that governs the production of bioactive compounds in McJXR, offering clear targets for future genetic engineering and targeted extraction strategies.

### 4.3 Flowers and leaves are the primary contributors to antioxidant activity in McJXR, with flavonoids and polyphenols serving as key bioactive constituents

A systematic evaluation of antioxidant capacity revealed that McJXR flowers exhibited the highest overall antioxidant activity, followed by leaves, with stems showing the lowest activity. This trend was highly consistent with the tissue-specific distribution of total flavonoid content (TFC) and total polyphenol content (TPC). Flowers displayed the highest TFC (10.82 mg RE/g DW), while leaves had the highest TPC (99.25 mg GAE/g DW), with both values significantly exceeding those in stems. In DPPH, ABTS, and FRAP assays, flower and leaf extracts consistently demonstrated strong free radical scavenging activity and reducing power. Although absolute values varied across assays due to differences in reaction mechanisms—hydrogen atom transfer (HAT) versus single electron transfer (SET) (Liang et al., 2022)—the consistent rank order of activity across tissues supports the robustness and reproducibility of the results.

Correlation analysis showed highly significant positive correlations between TFC/TPC and all three antioxidant indices (r > 0.94, p < 0.01), confirming that flavonoids and polyphenols are the principal determinants of McJXR’s antioxidant properties. Notably, flavonoids exhibited stronger associations with antioxidant activity (r > 0.94), suggesting their dominant role in radical neutralization. This is attributable to their molecular structure, including phenolic hydroxyl groups that act as hydrogen donors, conjugated π-systems that stabilize resulting radicals, and metal-chelating capacity that suppresses Fenton reactions (Shen et al., 2022). Specifically, luteolin and apigenin—identified as flower-enriched metabolites in this study—are well-documented potent antioxidants (Tian et al., 2021), and their preferential accumulation in flowers and leaves directly accounts for the enhanced antioxidant capacity observed in these tissues.

These findings have significant practical implications for the utilization of McJXR. While traditionally used as a whole-plant material, our data support a tissue-specific harvesting and processing strategy: prioritizing flowers and leaves can enhance the consistency of active ingredient content and improve product quality. Given the increasing demand for natural antioxidants in food, cosmetic, and nutraceutical industries (Xu et al., 2017), McJXR flower and leaf extracts represent promising alternatives to synthetic antioxidants such as BHA and BHT, thereby enabling high-value applications. Moreover, these results provide pharmacological validation of traditional medicinal practices, as metabolically active tissues—particularly leaves and flowers—are enriched in bioactive secondary metabolites.

### 4.4 Core metabolites associated with antioxidant activity in McJXR: identification and tissue-specific profiles

To identify key bioactive compounds underlying McJXR’s antioxidant properties, we performed Mantel tests correlating the top 20 VIP-ranked differential metabolites with ABTS, DPPH, and FRAP antioxidant indices. A set of marker metabolites exhibited significant positive correlations, providing direct biochemical evidence for the molecular basis of McJXR’s antioxidant capacity. Notably, several compounds showed consistently strong associations across multiple tissue comparisons (McJXR-S vs. McJXR-L; McJXR-S vs. McJXR-F), confirming their potential role as candidate functional components.

These compounds include volatile phenolics (thymol, carvacrol), whose phenolic hydroxyl groups are known to donate hydrogen atoms, thereby interrupting free radical chain reactions (Chroho et al., 2024; Rathod et al., 2021). Their strong positive correlations with ABTS and DPPH assays (r > 0.80) in the Mantel test suggest they may serve as key contributors to McJXR’s antioxidant activity, though this does not establish them as definitive core antioxidants. Terpenoids (e.g., humulene) and biogenic amines/amino acid derivatives (octopamine, GABA) also exhibited significant positive correlations with antioxidant indices, indicating potential roles in antioxidant defense—possibly through hydrophobic interactions (humulene) (Dalavaye et al., 2024) or via ROS scavenging and regulation of oxidative stress (octopamine, GABA) (Ayanlowo et al., 2020; Das and Goyal, 2015). It is important to emphasize that these interpretations are based solely on correlative analyses (Mantel test) between differentially abundant metabolites and antioxidant metrics, without direct experimental validation. Therefore, the functional contributions of these metabolites to McJXR’s antioxidant system remain hypothetical and require further confirmation through biochemical assays or targeted functional studies.

The Mantel test results further revealed tissue-specific patterns in antioxidant metabolism. For instance, p-coumaric acid—a key intermediate in phenylpropanoid biosynthesis with intrinsic antioxidant activity (Boo, 2019)—showed a significant correlation with FRAP only in the stem vs. flower comparison. Luteolin exhibited significant associations exclusively in floral tissues, consistent with flowers being the primary site of flavonoid biosynthesis in McJXR. This spatial differentiation indicates that McJXR’s overall antioxidant capacity arises from organ-specific metabolic strategies: flowers specialize in high-potency flavonoids (e.g., luteolin), whereas leaves accumulate a broader array of phenolic compounds (e.g., thymol, carvacrol) and biogenic amines (e.g., GABA). Such functional specialization is likely regulated by tissue-specific transcriptional networks, enabling each organ to fulfill distinct ecological and physiological roles.

Compared to conventional TFC/TPC-based correlation analyses, this study identifies potential molecular targets for the development of high-value products—such as thymol-enriched or GABA-rich extracts—and provides a theoretical foundation for future research aimed at enhancing the biosynthesis of key compounds through molecular breeding. However, a critical limitation must be acknowledged: the associations reported here are derived from untargeted metabolomic profiling and Mantel correlation analysis. The direct antioxidant effects, dose-response relationships, and underlying mechanisms of candidate metabolites (e.g., thymol, carvacrol, humulene) have not been validated through isolation, purification, or exogenous application bioassays of individual compounds. Consequently, current inferences regarding their contribution to antioxidant activity remain correlative rather than causal, and the designation of these metabolites as “core antioxidant compounds” would constitute an overinterpretation of the data. To address this limitation, future studies should conduct dose-response experiments and functional bioassays using purified individual metabolites to confirm their direct antioxidant activity and determine their effective concentrations.

## 5 Conclusion

This study integrated GC–MS and LC–MS/MS-based metabolomics profiling with standardized antioxidant activity assays to systematically elucidate the tissue-specific correlations between metabolite accumulation and antioxidant function in McJXR. The results demonstrate that flowers and leaves are the primary part of antioxidant-active component enrichment, where flavonoids, phenolic compounds, and characteristic metabolites such as thymol and luteolin collectively constitute the biochemical basis of its antioxidant activity. KEGG pathway enrichment analysis further revealed that key metabolic pathways—including phenylpropanoid biosynthesis, flavonoid biosynthesis, and ABC transporter pathways—play crucial regulatory roles in mediating the tissue-specific distribution of these bioactive constituents. These findings not only provide mechanistic insights into the metabolic basis of tissue-dependent antioxidant activity in McJXR but also establish a robust theoretical foundation for the precise, tissue-specific utilization of plant resources and the development of high-value antioxidant products.

## Data Availability

The original contributions presented in the study are included in the article/**Supplementary Material**. Further inquiries can be directed to the corresponding authors.
